# Evaluation of monocyte to HDL cholesterol ratio and other inflammatory markers in patients with psoriasis^☆^^☆☆^

**DOI:** 10.1016/j.abd.2020.02.008

**Published:** 2020-07-12

**Authors:** Mumtaz Cem Sirin, Selma Korkmaz, Ijlal Erturan, Basak Filiz, Buket Cicioglu Aridogan, Emel Sesli Cetin, Mehmet Yildirim

**Affiliations:** a Department of Medical Microbiology, Faculty of Medicine, Suleyman Demirel University, Isparta, Turkey; b Department of Dermatology, Faculty of Medicine, Suleyman Demirel University, Isparta, Turkey

**Keywords:** Biological markers, Cholesterol, HDL, C-Reactive protein, Inflammation, Monocytes, Psoriasis, Serum amyloid A protein

## Abstract

**Background:**

Psoriasis is a chronic systemic inflammatory disease frequently associated with serious comorbidities.

**Objectives:**

To investigate the systemic inflammatory burden in psoriasis and to assess the correlation between traditional and novel inflammatory markers and the severity of the disease.

**Methods:**

This cross-sectional study was conducted on 60 patients with psoriasis vulgaris and 50 healthy volunteers. Data including demographics, Psoriasis Area and Severity Index scores, and laboratory results were analyzed and compared.

**Results:**

Compared with the control group, the psoriatic patients had significantly higher high sensitive C-reactive protein, serum amyloid A, erythrocyte sedimentation rate, leukocyte, neutrophil, neutrophil-to-lymphocyte ratio, monocyte to high density lipoprotein (HDL) cholesterol ratio, and aspartate aminotransferase levels, and significantly lower HDL cholesterol levels (p < 0.05). No significant difference was found in procalcitonin, lymphocyte, monocyte, hemoglobin, red blood cell distribution width, platelet, mean platelet volume, platelet distribution width, lymphocyte-to-monocyte ratio, anti-cyclic citrullinated peptide, glucose, alanine aminotransaminase, blood urea nitrogen, creatinine, triglyceride, total cholesterol, and LDL cholesterol levels between the two groups (p > 0.05). The Psoriasis Area and Severity Index score was positively correlated with high-sensitivity C-reactive protein, serum amyloid A, and monocyte to HDL cholesterol ratio, and negatively correlated with lymphocyte-to-monocyte ratio (p < 0.05).

**Study limitations:**

This was a single-center study with relatively limited numbers of patients and controls.

**Conclusions:**

The data show that high sensitivity C-reactive protein, serum amyloid A, erythrocyte sedimentation rate, neutrophil-to-lymphocyte ratio, and monocyte to HDL cholesterol ratio can be used as markers of systemic inflammation in patients with psoriasis. Moreover, high sensitivity C-reactive protein, serum amyloid A, monocyte to HDL cholesterol ratio and lymphocyte-to-monocyte ratio are closely related to the Psoriasis Area and Severity Index score, and they may be regarded as objective indicators in determining the disease severity.

## Introduction

Psoriasis is a chronic immune-mediated inflammatory skin disease affecting approximately 1 %–3 % of the general population.^1^, ^2^, ^3^ The disease primarily involves the skin; however, it is considered that psoriasis is a systemic disorder frequently associated with serious comorbidities such as cardiovascular diseases, metabolic syndrome, arthritis, glucose intolerance, and obesity.^3^, ^4^, ^5^ The disease process in the skin is proposed to be histopathologically characterized by impaired differentiation and hyperproliferation of epidermal keratinocytes and infiltration of immune cells, predominantly T-cells and dendritic cells. Inflammation is generally attributed to the activation of circulating neutrophils and monocytes mediated by proinflammatory cytokines. The pathological events in the skin ultimately lead to the local formation of psoriatic plaques, and further contribute to the development of persistent systemic inflammation.^1^, ^6^, ^7^, ^8^, ^9^, ^10^

Systemic inflammation can be assessed by using various biochemical and hematological markers. However, there have not yet been any sensitive, specific, and clinically useful biomarkers indicating disease activity for psoriasis.^8^, ^11^ Biomarkers are important in clinical practice because they provide objective and quantitative assessment of diagnosis, disease processes, and therapy response.^7^ Currently, the most commonly used scale in determining psoriasis severity is the Psoriasis Area and Severity Index (PASI) score, which is based on a visual evaluation of the skin lesions and combines the severity (erythema, induration, and desquamation) and percentage of affected area. But this method is limited due to its subjective quality and has restricted utility for non-plaque-type psoriasis.^5^, ^8^, ^9^, ^12^

Among the acute phase reactants, C-reactive protein (CRP) and erythrocyte sedimentation rate (ESR) are two well-recognized inflammatory indices and can be used to assess the burden of psoriatic inflammation and disease activity in patients with psoriasis.^1^, ^3^, ^10^, ^12^ Moreover, CRP elevation is regarded as an independent risk factor for cardiovascular disease.^12^ Serum amyloid A (SAA), another important acute phase reactant, is also accepted as an apolipoprotein relevant in cholesterol metabolism and has been associated with atherogenesis and cardiovascular disease.^4^, ^13^ Procalcitonin (PCT), the precursor peptide of calcitonin hormone, is released in response to inflammation and has gained widespread interest, especially as a biomarker of bacterial infection.^14^, ^15^, ^16^

The investigation of other inflammatory markers such as cytokines, adhesion molecules, and chemokines by flow cytometric assays is not available for the majority of general hospitals and health centers, due to high costs, and long and laborious procedures.^5^, ^8^, ^16^ In recent years, hematological parameters such as the neutrophil-to-lymphocyte ratio (NLR), the lymphocyte-to-monocyte ratio (LMR), and monocyte-to-high density lipoprotein cholesterol ratio (MHR) have been investigated in different systemic diseases as indicators of inflammation.^5^, ^8^, ^17^, ^18^, ^19^, ^20^ These are readily available and cost-effective parameters that can be obtained by routine complete blood count (CBC) and biochemical analysis.

The aim of the present study was to investigate the systemic inflammatory burden in psoriasis by using different biochemical and hematological markers and to evaluate the correlation between these markers and the severity of the disease.

## Methods

This cross-sectional study was carried out at the Department of Dermatology in collaboration with the Department of Medical Microbiology, Suleyman Demirel University Research and Practice Hospital, between March 2019 and September 2019. Ethical approval was obtained from the Ethics Committee of Suleyman Demirel University, Faculty of Medicine (document No. 72867572.050.01.04/39475). The research protocol was conducted in accordance with the Helsinki Declaration, and written informed consent was taken from all the participants before the study commenced.

Patients diagnosed clinically and histopathologically with psoriasis vulgaris were enrolled in the study. Patients with any other inflammatory skin disorders; severe cardiovascular disease; chronic hepatic or renal diseases; autoimmune disorders, neoplasms, endocrine disorders, and hematologic diseases; or any history of taking systemic or topical medication within the last three months were excluded. The control group consisted of healthy age- and sex-matched volunteers with no history of systemic disease.

The dermatologic examinations and PASI scoring were performed by the same dermatologist. Age, gender, family history, age of onset, disease duration, and PASI values of each patient were recorded.

Venous blood samples were taken from each subject, and then centrifuged prior to testing. The serum levels of SAA were measured by nephelometric method (BN II analyzer, Siemens Healthcare Diagnostics – Erlangen, Germany). The serum PCT and anti-cyclic citrullinated peptide (anti-CCP) antibody levels were analyzed by using an automated chemiluminescence immunoassay method (Roche Cobas e601 analyzer; Roche Diagnostics – Mannheim, Germany). Biochemical analysis (high sensitivity C-reactive protein [hsCRP], fasting blood glucose, alanine aminotransaminase [ALT], aspartate aminotransferase [AST], blood urea nitrogen [BUN], creatinine, fasting triglyceride [TG], total cholesterol [TC], low density lipoprotein cholesterol [LDL-C], and high density lipoprotein cholesterol [HDL-C]) was performed using an AU 5800 chemistry analyzer (Beckman Coulter – Brea, California, United States). Complete blood count analysis (leukocyte count [WBC], neutrophil count, lymphocyte count, monocyte count, hemoglobin [Hb], red blood cell distribution width [RDW], platelet count [PLT], mean platelet volume [MPV], and platelet distribution width [PDW]) and erythrocyte sedimentation rate (ESR) measurement were performed using an UniCel DxH 800 hematology analyzer (Beckman Coulter – Brea, California, United States), and a Test-1 analyzer (Alifax – Padova, Italy), respectively. For CBC and ESR analyses, venous blood samples taken from each patient were collected in blood tubes containing ethylenediaminetetraacetic acid or citrate. All assays were performed according to the manufacturers’ instructions. On the basis of biochemical analysis and CBC data, the values of NLR, LMR, and MHR were calculated.

Statistical analysis was performed using SPSS v. 20 (SPSS Inc. – Chicago, Illinois, United States). The Kolmogorov-Smirnov test was used to evaluate the normality of the data. Accordingly, Student’s *t*-test or the Mann-Whitney *U*-test was used to compare the differences in continuous variables between groups. The chi-squared test was used for categorical variables. Results were expressed as frequencies and percentages, or mean ± Standard Deviation. A receiver operating characteristic (ROC) curve was drawn for the MHR, and the area under the curve (AUC) value with 95% confidence interval (CI) was calculated. A cut-off value was determined for predicting psoriasis, and sensitivity, specificity, positive predictive value (PPV), and negative predictive value (NPV) were calculated. The correlations between variables were evaluated by Spearman correlation analysis. A *p*-value of < 0.05 was considered statistically significant.

## Results

Sixty patients with psoriasis and 50 healthy controls were included in the study. Comparison of demographic characteristics (age, gender) and laboratory findings in the patient and control groups are shown in table 1. There was a family history of psoriasis in 31.7% (n = 19) of the patients. The mean ages of onset and disease duration in patients with psoriasis were 29.65 ± 13.85 years (range 8‒63) and 9.14 ± 7.81 years (range 1‒31), respectively. The mean PASI score was 10.2 ± 9.56 (range 0.8‒46.8) in the patient group.

**Table 1:** Comparison of demographic characteristics and laboratory findings in the patient and control groups.

	Patient group (n = 60)	Control group (n = 50)	*p*-value
Age (years)	37.91 ± 13.46	34.66 ± 10.41	0.165
Gender (M/F)	31 (51.7) / 29 (48.3)	25 (50) / 25 (50)	0.862
hsCRP (mg/L)	6.2 ± 12.15	1.74 ± 0.76	**0.025**
SAA (mg/L)	28.55 ± 56.67	10.53 ± 10.28	**0.029**
PCT (ng/mL)	0.0256 ± 0.012	0.0246 ± 0.005	0.085
ESR (mm/hr)	24.46 ± 18.42	10.44 ± 8.65	**< 0.001**
WBC (×10^3^/µL)	7.68 ± 2.58	6.8 ± 1.51	**0.036**
Neutrophils (x×10^3^/µL)	4.77 ± 2.34	3.94 ± 1.25	**0.026**
Lymphocytes (x10^3^/µL)	2.12 ± 0.64	2.15 ± 0.49	0.806
Monocytes (×10^3^/µL)	0.56 ± 0.19	0.51 ± 0.11	0.102
Hb (g/dL)	14.32 ± 1.74	14.77 ± 1.66	0.166
RDW (%)	14.11 ± 1.46	13.8 ± 1.91	0.346
PLT (×10^3^/µL)	249.81 ± 59.42	248.16 ± 53.11	0.879
MPV (fL)	8.71 ± 0.83	8.63 ± 0.78	0.643
PDW (%)	16.8 ± 0.43	16.77 ± 0.42	0.754
NLR	2.42 ± 1.51	1.9 ± 0.7	**0.009**
LMR	4 ± 1.36	4.3 ± 1.14	0.223
MHR	12.21 ± 4.94	10.1 ± 3.11	**0.010**
Anti-CCP (U/mL)	8.58 ± 8.94	7.11 ± 0.03	0.248
Glucose (mg/dL)	105.06 ± 45.22	93.63 ± 17.38	0.095
ALT (IU/L)	24.85 ± 19.2	20.92 ± 11.75	0.209
AST (IU/L)	26.52 ± 14.97	21.75 ± 6.27	**0.038**
BUN (mg/dL)	13.11 ± 3.55	12.32 ± 2.86	0.204
Creatinine (mg/dL)	0.91 ± 0.15	0.95 ± 0.16	0.105
Triglycerides (mg/dL)	129.98 ± 62.24	122.97 ± 57.73	0.545
Total cholesterol (mg/dL)	186.33 ± 29.07	192.35 ± 33.09	0.160
LDL-C (mg/dL)	111.95 ± 26.47	113.89 ± 29.01	0.715
HDL-C (mg/dL)	48.37 ± 9.39	53.21 ± 10.52	**0.020**

Values are expressed as n (%) or mean ± SD.

hsCRP, high sensitivity C-reactive protein; SAA, serum amyloid A; PCT, procalcitonin; ESR, erythrocyte sedimentation rate; WBC, leukocyte count; Hb, hemoglobin; RDW, red blood cell distribution width; PLT, platelet count; MPV, mean platelet volume; PDW, platelet distribution width; NLR, neutrophil-to-lymphocyte ratio; LMR, lymphocyte-to-monocyte ratio; MHR, monocyte to high density lipoprotein ratio; anti-CCP, anti-cyclic citrullinated peptide; ALT, aminotransaminase; AST, aspartate aminotransferase; BUN, blood urea nitrogen; LDL-C, low density lipoprotein cholesterol; HDL-C, high density lipoprotein cholesterol.

Bold values signifies Statistically significant values.

There were significant differences in hsCRP, SAA, ESR, WBC, neutrophil, NLR, MHR, AST, and HDL-C levels between the patient and control groups (p < 0.05). Compared with the control group, the psoriatic patients had significantly higher hsCRP, SAA, ESR, WBC, neutrophil, NLR, MHR, and AST levels, and significantly lower HDL-C levels (Table 1).

No significant difference was found in PCT, lymphocyte, monocyte, Hb, RDW, PLT, MPV, PDW, LMR, anti-CCP, glucose, ALT, BUN, creatinine, TG, TC, and LDL-C levels between the two groups. Anti-CCP was increased above the cut-off level (≥ 17 U/mL) in three out of 60 psoriatic patients, while all subjects in the control group had normal anti-CCP levels. Among the three patients, only one patient fulfilled the CASPAR criteria for the diagnosis of psoriatic arthritis.

Psoriatic patients were divided into two groups according to their PASI score. The severity of the disease based on the PASI score was classified as mild (PASI < 10) and moderate-severe (PASI ≥ 10). Thirty-seven patients had mild psoriasis, while 23 patients had moderate-severe psoriasis. Comparison of laboratory findings in psoriatic patients with PASI < 10 and PASI ≥ 10 is shown in table 2. Compared with patients with PASI < 10, patients with PASI ≥ 10 had significantly higher hsCRP, MHR, and monocyte levels, and significantly lower LMR and HDL-C levels (p < 0.05).

**Table 2:** Comparison of laboratory findings in psoriatic patients with PASI < 10 and PASI ≥ 10.

	PASI < 10 (n = 37)	PASI ≥ 10 (n = 23)	*p*-value
hsCRP (mg/L)	3.66 ± 5.07	10.3 ± 18.03	**0.038**
SAA (mg/L)	22.27 ± 43.15	38.66 ± 73.42	0.280
PCT (ng/mL)	0.0247 ± 0.012	0.0271 ± 0.012	0.474
ESR (mm/hr)	23.1 ± 18.09	26.65 ± 19.13	0.473
WBC (×10^3^/µL)	7.22 ± 1.75	8.43 ± 3.46	0.080
Neutrophils (×10^3^/µL)	4.35 ± 1.33	5.43 ± 3.32	0.081
Lymphocytes (×10^3^/µL)	2.12 ± 0.63	2.12 ± 0.67	0.996
Monocytes (×10^3^/µL)	0.52 ± 0.15	0.63 ± 0.21	**0.019**
Hb (g/dL)	14.33 ± 1.52	14.3 ± 2.08	0.945
RDW (%)	14.04 ± 1.4	14.21 ± 1.59	0.664
PLT (×10^3^/µL)	250.27 ± 55.43	249.08 ± 66.64	0.941
MPV (fL)	8.71 ± 0.93	8.69 ± 0.66	0.917
PDW (%)	16.79 ± 0.47	16.81 ± 0.36	0.874
NLR	2.17 ± 0.77	2.8 ± 2.22	0.118
LMR	4.38 ± 1.54	3.4 ± 0.67	**0.007**
MHR	10.71 ± 3.9	14.63 ± 5.54	**0.003**
Anti-CCP (U/mL)	7.47 ± 2.3	10.35 ± 14.16	0.229
Glucose (mg/dL)	99.02 ± 21.25	114.78 ± 67.7	0.192
ALT (IU/L)	23.85 ± 21.04	26.46 ± 16.11	0.613
AST (IU/L)	27.23 ± 17.96	25.36 ± 8.42	0.642
BUN (mg/dL)	13.48 ± 3.19	12.52 ± 4.07	0.311
Creatinine (mg/dL)	0.92 ± 0.14	0.88 ± 0.16	0.296
Triglycerides (mg/dL)	130.07 ± 62.27	129.84 ± 63.6	0.989
Total cholesterol (mg/dL)	191.81 ± 26.78	177.52 ± 31	0.064
LDL-C (mg/dL)	115.34 ± 26.09	106.51 ± 26.73	0.212
HDL-C (mg/dL)	50.44 ± 8.86	45.03 ± 9.43	**0.017**

Values are expressed as mean ± SD.

hsCRP, high sensitivity C-reactive protein; SAA, serum amyloid A; PCT, procalcitonin; ESR, erythrocyte sedimentation rate; WBC, leukocyte count; Hb, hemoglobin; RDW, red blood cell distribution width; PLT, platelet count; MPV, mean platelet volume; PDW, platelet distribution width; NLR, neutrophil-to-lymphocyte ratio; LMR, lymphocyte-to-monocyte ratio; MHR, monocyte to high density lipoprotein ratio; anti-CCP, anti-cyclic citrullinated peptide; ALT, aminotransaminase; AST, aspartate aminotransferase; BUN, blood urea nitrogen; LDL-C, low density lipoprotein cholesterol; HDL-C, high density lipoprotein cholesterol.

Bold values signifies Statistically significant values.

Correlations between inflammation-related laboratory parameters and PASI scores in psoriatic patients are presented in table 3. PASI score was positively correlated with hsCRP (r = 0.255, p = 0.049), SAA (r = 0.257, p = 0.047), and MHR (r = 0.323, p = 0.012), and negatively correlated with LMR (r = -0.313, p = 0.015). There were no correlations between PASI score and other inflammation-related laboratory parameters (PCT, ESR, NLR, anti-CCP, WBC, neutrophils, lymphocytes, monocytes, RDW, PLT, MPV, and PDW) (p > 0.05).

**Table 3:** Correlation between inflammation-related laboratory parameters and PASI score in psoriatic patients (r-value [*p*-value]).

	PASI	hsCRP	SAA	PCT	ESR	NLR	LMR	MHR	Anti-CCP
hsCRP	**0.255 (0.049)**	‒	‒	‒	‒	‒	‒	‒	‒
SAA	**0.257 (0.047)**	**0.396 (0.002)**	‒	‒	‒	‒	‒	‒	‒
PCT	0.038 (0.774)	0.231 (0.076)	0.039 (0.766)	‒	‒	‒	‒	‒	‒
ESR	0.012 (0.929)	**0.433 (0.001)**	0.220 (0.091)	0.150 (0.253)	‒	‒	‒	‒	‒
NLR	0.239 (0.066)	0.196 (0.133)	0.242 (0.062)	0.016 (0.905)	0.066 (0.615)	‒	‒	‒	‒
LMR	**-0.313 (0.015)**	0.007 (0.955)	0.011 (0.934)	-0.087 (0.510)	0.058 (0.657)	**-0.532 (<0.001)**	‒	‒	‒
MHR	**0.323 (0.012)**	0.145 (0.269)	-0.138 (0.294)	**0.257 (0.047)**	-0.094 (0.476)	0.117 (0.374)	**-0.524 (<0.001)**	‒	‒
Anti-CCP	0.065 (0.620)	0.055 (0.675)	-0.069 (0.598)	-0.035 (0.793)	0.128 (0.331)	-0.035 (0.793)	-0.193 (0.140)	0.180 (0.170)	‒
WBC	0.170 (0.193)	0.085 (0.517)	0.032 (0.806)	**0.344 (0.007)**	-0.027 (0.838)	0.234 (0.072)	-0.081 (0.539)	**0.521 (<0.001)**	0.096 (0.464)
Neutrophils	0.214 (0.100)	0.137 (0.296)	0.141 (0.282)	**0.267 (0.039)**	-0.113 (0.390)	**0.641 (<0.001)**	-0.252 (0.053)	**0.419 (0.001)**	0.065 (0.623)
Lymphocytes	-0.049 (0.711)	-0.040 (0.762)	-0.132 (0.316)	0.220 (0.092)	0.020 (0.880)	**-0.587 (<0.001)**	**0.462 (<0.001)**	**0.259 (0.045)**	0.075 (0.568)
Monocytes	0.233 (0.073)	-0.004 (0.977)	-0.129 (0.327)	**0.292 (0.023)**	0.013 (0.919)	0.047 (0.721)	**-0.607 (<0.001)**	**0.813** **(<0.001)**	0.242 (0.062)
RDW	0.120 (0.360)	**0.299 (0.020)**	0.137 (0.298)	-0.083 (0.527)	**0.442 (<0.001)**	0.041 (0.755)	-0.060 (0.649)	0.012 (0.925)	-0.117 (0.371)
PLT	-0.095 (0.469)	0.084 (0.523)	-0.001 (0.992)	0.207 (0.113)	**0.313 (0.015)**	**0.298 (0.021)**	-0.154 (0.240)	0.150 (0.251)	**0.317 (0.014)**
MPV	0.121 (0.357)	0.034 (0.799)	0.237 (0.069)	-0.126 (0.339)	0.048 (0.714)	-0.144 (0.271)	0.144 (0.274)	-0.115 (0.380)	0.015 (0.909)
PDW	0.211 (0.106)	0.226 (0.083)	0.130 (0.324)	-0.101 (0.442)	0.033 (0.800)	-0.118 (0.371)	0.103 (0.432)	-0.148 (0.260)	0.041 (0.755)

hsCRP, high sensitivity C-reactive protein; SAA, serum amyloid A; PCT, procalcitonin; ESR, erythrocyte sedimentation rate; WBC, leukocyte count; Hb, hemoglobin; RDW, red blood cell distribution width; PLT, platelet count; MPV, mean platelet volume; PDW, platelet distribution width; NLR, neutrophil-to-lymphocyte ratio; LMR, lymphocyte-to-monocyte ratio; MHR, monocyte to high density lipoprotein ratio.

Bold values signifies Statistically significant values.

The optimal cut-off value of MHR for the prediction of psoriasis was determined by using ROC curve analysis (AUC = 0.631, 95% CI: 0.526‒0.735). The cut-off value of ≥ 9.52 had diagnostic sensitivity of 71.7% (95% CI: 59.2‒81.5), diagnostic specificity of 54% (95% CI: 40.4‒67), PPV of 65.2% (95% CI: 53.1‒75.5), and NPV of 61.4% (95% CI: 46.6‒74.3) in predicting psoriasis (data not shown).

## Discussion

To date, there have been numerous investigations on biomarkers in psoriasis; however, there have not yet been any clinically relevant biomarkers which are specific for psoriasis.^1^, ^11^ The PASI scoring and a careful clinical examination still remain the mainstay of severity evaluation of psoriasis.^12^ Nonetheless, some inflammatory markers, such as CRP and ESR, are still being used in daily practice with PASI score and are helpful in diagnosis and clinical follow-up, to some degree.^1^, ^3^, ^12^ However, there is still need for more specific, inexpensive, practical, and reliable methods for the determination of psoriasis activity. In this study, besides well-known serum inflammatory markers such as hsCRP, SAA, and PCT, the authors also aimed to investigate novel inflammatory markers that can easily be obtained from routine CBC and biochemical analysis.

CRP, the most commonly used serum inflammation marker, can be routinely measured by the high sensitivity assays.^6^, ^12^ Sensitive CRP measurements can determine even lower levels of CRP, which are significantly related to certain inflammatory diseases and cardiovascular diseases.^4^ Many studies have reported the clinical utility of hsCRP in psoriasis.^4^, ^6^, ^9^, ^10^, ^12^, ^21^ In the present study, hsCRP levels were significantly higher in patients than in controls and were positively correlated with the PASI score and ESR (Table 3). The current data support the previous reports indicating that serum CRP level can be used as an appropriate marker for assessing severity of disease in psoriasis.

In contrast to CRP or ESR, less is known about the clinical usefulness of SAA in psoriasis. In addition, compared with CRP, higher costs limit the widespread usage of SAA, especially in low-resource countries. Some reports have shown the association between SAA and psoriasis.^4^, ^13^, ^22^, ^23^ Morizane et al.^13^ suggested that psoriatic skin inflammation enhances SAA expression, and the elevation of serum SAA levels may lead to the development of systemic complications such as atherosclerosis in psoriasis patients. Dogan et al.^22^ reported that SAA is a more specific marker than CRP for assessing systemic inflammation in psoriatic patients. In the present study, SAA levels were found to be significantly higher in patients than in controls and positive correlations were noted between SAA, hsCRP, and PASI score (Table 3). SAA and hsCRP are the most sensitive indicators for inflammation, and the parallel use of these two parameters may increase the sensitivity and accuracy in the evaluation of disease activity and severity in psoriasis.

Many clinical studies have indicated that PCT measurement is useful for the diagnosis and monitoring of severe bacterial infections, sepsis, and multiple organ dysfunction.^14^, ^15^, ^16^ Not surprisingly, the current study found normal PCT values in patients with psoriasis. In contrast, Ibrahimbas et al.^14^ reported significantly increased levels of serum PCT in patients who had chronic plaque psoriasis with exacerbations. They proposed that a bacterial antigenic stimulus might lead to this elevation. In accordance with the current results, Nagai et al.^15^ found no significant elevation in PCT levels in patients with psoriasis vulgaris, psoriatic arthritis, and generalized pustular psoriasis. Those authors suggested that a microbial infection is unlikely in the pathogenesis of psoriasis. Large prospective studies with carefully selected cases with different forms of psoriasis are needed to clarify the role of PCT in psoriasis.

Citrullination is a post-translational modification of proteins that occurs in the context of inflammation.^24^ Anti-CCP antibodies are directed against citrullinated peptides and are commonly used as a diagnostic marker for rheumatoid arthritis.^24^, ^25^ However, recent reports suggested that anti-CCP antibodies have also been observed in psoriatic arthritis, juvenile idiopathic arthritis, gout, and ankylosing spondylitis.^25^, ^26^ Furthermore, it has been demonstrated that there are citrullinated epitopes within atherosclerotic plaques that are targeted by anti-CCP antibodies and that anti-CCP elevation is correlated with progressively increased cardiovascular risk.^24^ In light of these observations, the present study aimed to analyze anti-CPP levels in terms of evaluating the inflammatory burden in psoriasis. It was found that higher anti-CCP levels occurred in psoriatic patients when compared with the control group. Elevated anti-CCP levels were also observed in patients with moderate-severe psoriasis when compared with the patients with mild psoriasis. Although not reaching statistical significance, it may be speculated that these results partially reflect the difference in the inflammatory burden between the groups.

Immunocompetent white blood cells, including neutrophils, lymphocytes, and monocytes, play a critical role in the systemic inflammatory response.^16^ Neutrophils are among the first cells to migrate to sites of inflammation and are also fairly active in recruiting other immune cells into the lesions, such as monocyte-derived macrophages.^9^ The systemic inflammatory reaction typically leads to neutrophilia and relative lymphocytopenia.^16^ NLR, a combined index using neutrophil and lymphocyte counts, can more accurately reflect fluctuations between neutrophils and lymphocytes in systemic inflammation.^8^, ^16^ In recent years, NLR has been shown to be a marker of systemic inflammation in many diseases such as atherosclerosis, myocardial infarction, diabetes mellitus, ulcerative colitis, malignancies, and psoriasis.^3^, ^5^, ^8^, ^17^^,^^18^, ^21^, ^27^, ^28^ LMR, which also reflects the burden of systemic inflammation, has been widely investigated as a useful prognostic marker in various cancers.^19^ No published data were found reporting the relationship between psoriasis and LMR.

The present study found a significantly higher WBC count in psoriatic patients owing to increased neutrophils and monocytes counts. Similar to previous studies, NLR was significantly higher in patients with psoriasis compared to controls.^3^, ^5^, ^8^, ^17^, ^18^, ^21^, ^27^, ^28^ However, in the literature, discrepant results were reported about the correlation between NLR values and PASI score. Kim et al.^3^ demonstrated a positive correlation between NLR and PASI score. However, the current results were consistent with the results of two studies conducted in Turkey, suggesting that no correlation was found between NLR and PASI score.^8^, ^28^ These discrepancies may be due to the differences in sample size, study population, or mean PASI values. Interestingly, a significant difference was not found between the patient and control groups in terms of LMR values, but significantly different LMR values was observed between mild psoriatic patients and moderate-severe psoriatic patients, and significant negative correlations were noted between LMR, NLR, and PASI score (Table 3). Based on these results, it may be noted that NLR is more useful in demonstrating inflammation in patients with psoriasis, and LMR can be preferred to evaluate the disease activity in psoriasis. Nevertheless, the psoriatic patients with high NLR or low LMR values should be carefully monitored even if their PASI scores are low.

Platelets have a crucial role in maintaining homeostasis, but are also regarded as important mediators of acute and chronic inflammatory reactions. They may assist other inflammatory cells to migrate to the lesion sites by releasing large amounts of inflammatory cytokines, and ultimately create an inflammatory environment.^2^, ^11^, ^28^ MPV and PDW are known as PLT activation indices, reflecting the PLT production rate and stimulation.^2^, ^11^, ^29^ In the literature, there are contradictory results about the relationship between platelet activation markers and psoriasis.^2^, ^27^, ^29^, ^30^ In a study carried out in Korea, significantly higher MPV and PDW values were found in patients with psoriasis than in controls and a positive correlation was observed between MPV and PASI score. ^11^ In contrast, Saleh et al.^31^ reported that there was no significant difference in MPV levels between patients with psoriasis and controls. Likewise, the present study found higher platelet, MPV, and PDW levels in psoriatic patients compared to control group, but the difference between the two groups was not significant.

Monocytes and macrophages have a key role in the inflammation process during the development and progression of atherosclerosis. In the early stage of the process, circulating monocytes migrate to the subendothelial space of the arterial wall, mature into macrophages, and internalize oxidized LDLs and other lipids. Then, these cells differentiate into the foam cells to release proinflammatory cytokines at the site of inflammation that activate T-lymphocytes, platelets, and further monocytes.^20^, ^32^ However, HDL-C inhibits monocyte activities, impedes the transformation of monocytes to macrophages, and eliminates cholesterol from these cells, which leads to a restricted inflammatory response.^20^, ^32^, ^33^, ^34^ For this reason, it is reasonable to combine these two parameters into a single index (MHR) as an inflammatory marker. It has been reported that increased MHR level was associated with systemic inflammation and MHR could be used as predictive marker of future cardiovascular disease.^20^, ^32^, ^33^, ^34^, ^35^ To the best of the present authors’ knowledge, MHR has not yet been evaluated in patients with psoriasis. The current study revealed that MHR levels in the patient group were higher than those of the healthy controls, and that MHR values were positively correlated with PASI score. As practical, cost-effective, reproducible parameter of the CBC and biochemical analysis, MHR can be used in clinical practice for the evaluation of disease severity in psoriasis. The evaluation of MHR levels together with the other markers that predict cardiovascular risk such as hsCRP and SAA may provide a novel basis for the identification of psoriatic patients at increased risk of cardiovascular disease. Nevertheless, further prospective studies with larger sample sizes are needed to validate the clinical use of MHR in psoriatic patients.

The present study has some limitations that should be considered when interpreting the results. First, this was a single-center study and had relatively limited numbers of patients and controls. Second, the inflammatory markers were measured only on admission, and it was not possible to evaluate the alterations in the levels of markers and PASI scores after the therapy during the follow-up period.

## Conclusions

In conclusion, the data demonstrate that increased hsCRP, SAA, ESR, NLR, and MHR levels can reflect the systemic inflammatory burden in psoriatic patients. Furthermore, hsCRP, SAA, MHR, and LMR are closely related to the PASI score and these markers can be used in daily practice to assess disease severity in psoriasis. However, additional studies are required to support these findings.

## Financial support

None declared.

## Authors’ contributions

Mumtaz Cem Sirin: Statistical analysis; approval of final version of the manuscript; conception and planning of the study; drafting and editing of the manuscript.

Selma Korkmaz: Statistical analysis; collection, analysis, and interpretation of data; critical review of the literature.

Ijlal Erturan: Statistical analysis; collection, analysis, and interpretation of data; critical review of the literature.

Basak Filiz: Collection, analysis, and interpretation of data; critical review of the literature.

Buket Cicioglu Aridogan: Approval of final version of the manuscript; participation in the design of the study; critical review of the manuscript.

Emel Sesli Cetin: Approval of final version of the manuscript; participation in the design of the study; critical review of the manuscript.

Mehmet Yildirim: Approval of final version of the manuscript; conception and planning of the study; critical review of the manuscript.

## Conflicts of interest

None declared.
